# Ozurdex® (a slow-release dexamethasone implant) in proliferative vitreoretinopathy: study protocol for a randomised controlled trial

**DOI:** 10.1186/1745-6215-14-358

**Published:** 2013-10-28

**Authors:** Philip J Banerjee, Catey Bunce, David G Charteris

**Affiliations:** 1 Moorfields Eye Hospital NHS Foundation Trust, City Road, EC1V 2PD London, UK; 2 Moorfields NIHR Clinical Research Facility, London, UK

## Abstract

**Background:**

Proliferative vitreoretinopathy (PVR) is the commonest cause of late anatomical failure in rhegmatogenous retinal detachment. Visual and anatomical outcomes remain poor despite advances in vitreoretinal surgical techniques with reported primary failure rates of up to nearly 50%. Numerous adjunctive medications have been evaluated in clinical trials with no agent gaining widespread acceptance and use.

This study was designed to investigate the benefits of using a slow-release dexamethasone implant delivered intra-operatively in patients undergoing vitrectomy surgery for retinal detachment with established PVR.

**Methods/design:**

For the study, 140 patients requiring vitrectomy surgery with silicone oil for retinal detachment with established PVR will be randomised to receive either standard treatment or study treatment in a 1:1 treatment allocation ratio. Both groups will receive the standard surgical treatment appropriate for their eye condition and routine peri-operative treatment and care, differing only in the addition of the supplementary adjunctive agent in the treatment group. The investigated primary outcome measure is stable retinal reattachment with removal of silicone oil without additional vitreoretinal surgical intervention at 6 months.

**Discussion:**

This is the first randomised controlled clinical trial to investigate the use of an adjunctive slow-release dexamethasone implant in patients undergoing vitrectomy surgery for retinal detachments with proliferative vitreoretinopathy.

**Trial registration:**

EudraCT No: 2011-004498-96.

## Background

Proliferative vitreoretinopathy (PVR) is the most common cause of late anatomic failure in retinal detachment surgery and is generally regarded as having an incidence of 5% to 11% of all rhegmatogenous retinal detachments [1]. PVR can be considered a maladapted wound healing response in the retina, which results in the formation of fibrocellular membranes on both surfaces of the retina and the posterior hyaloid face. Contraction of these membranes can result in distortion of the normal retinal topography with visually detrimental sequelae, and/or tractional retinal detachment, with the reopening of pre-existing breaks or the formation of new ones.

PVR represents a difficult vitreoretinal surgical challenge and despite the improvements in vitreoretinal surgery, a significant number of cases fail to achieve reattachment. Multiple surgeries are frequently required to eventually achieve final retinal attachment with poor visual results and unsatisfactory binocular visual outcomes [2-4]. Additionally, PVR management is costly in patient time and health-care resources [3].

Numerous adjunctive medications have been previously evaluated in clinical trials [5-12], yet no effective and safe adjunct has gained widespread acceptance to improve surgical and visual outcomes. The proposed study is a large-scale prospective randomised controlled clinical trial to determine the efficacy of Ozurdex as an adjunctive medication. A positive result of the study would have an immediate clinical application worldwide.

### Rationale

PVR peri-retinal membranes comprise four categories of cells; retinal pigment epithelium, glial, inflammatory and fibroblastic cells. These cells proliferate and may also be contractile and are thus targets for anti-proliferative agents. There is also a notable inflammatory component to the PVR process with marked blood-retinal barrier breakdown and intraocular fibrin formation. Thus both cellular proliferation and the intraocular inflammatory response are realistic targets for adjunctive treatments in PVR.

Steroid treatment can potentially influence both the inflammatory and proliferative components of PVR. Experimental work has substantiated the theory that corticosteroids can reduce Müller cell proliferation [13] and reduce the severity of PVR [14-16].

Previous small-scale, uncontrolled clinical studies of PVR have suggested intravitreal crystalline cortisone was well tolerated in PVR cases undergoing vitrectomy [17] and that systemic prednisolone [18], infused dexamethasone [19] and intravitreal triamcinolone [20] may reduce the severity of PVR although none of these studies were of sufficient power to provide a definitive answer. A small prospective non-comparative clinical study [21] concluded that intravitreal triamcinolone may have some benefit as an adjunct in non-trauma-related established PVR; however, this was contradicted by a small randomised controlled trial [22] where no additional benefit in surgical outcome was found. Both groups concluded that further larger studies were required to answer the question definitively.

The relatively short therapeutic duration of action of triamcinolone may offer an explanation as to why it has not emerged as a definitive adjunct, and the adoption of a longer-acting sustained-release preparation may offer additional advantages.

### Investigational medicinal product

Ozurdex® is an intravitreal implant containing 700 μg of dexamethasone in a slow-release preparation. It is indicated for the treatment of adult patients with macula oedema following retinal vein occlusion (either branch or central) [23], and inflammation of the posterior segment of the eye presenting as non-infectious uveitis [24]. It is increasingly being administered to treat the aforementioned retinal conditions and has a good local and systemic safety profile [23,24]. It is particularly useful in patients who have isolated ocular disease especially unilateral, providing an anti-inflammatory and anti-proliferative efficacy equal to or greater than that achieved with systemic administration while avoiding the unwanted systemic side effects of steroid use.

A study comparing the pharmacokinetics of this dexamethasone preparation in vitrectomised and non-vitrectomised experimental eyes concluded there was a similar vitreoretinal pharmacokinetic profile [25]. The clinical safety profile and therapeutic benefit of the investigational medicinal product (IMP) in patients who have previously undergone vitrectomy surgery has been reported [26-28]. The IMP reaches a therapeutic concentration shortly after administration and peaks at 2 months, thus adequately covering the active PVR process, proffering additional advantages over shorter-acting agents.

### Objectives

#### Primary objective

The primary objective is to test the hypothesis that an adjunctive slow-release dexamethasone implant, given at the time of surgery, can improve the anatomical outcome of vitreoretinal surgery for established PVR and that there will be stable retinal reattachment with removal of silicone oil without additional vitreoretinal surgical intervention at 6 months.

#### Secondary objectives

The secondary objectives are to determine whether an adjunctive slow-release dexamethasone implant, given at the time of surgery, has an effect on the following at 6 and 12 months following the primary study vitrectomy:

•visual acuity (Early Treatment Diabetic Retinopathy Study (ETDRS) method)

•macula oedema and thickness (spectralis domain optical coherence tomography (SD-OCT) analysis)

•development of overt PVR recurrence at any time point

•complete retinal reattachment

•posterior (post equatorial) retinal reattachment

•tractional retinal detachment

•hypotony or raised intraocular pressure (IOP)

•macula pucker or epiretinal membrane (ERM)

•cataract

•quality of life as assessed by NEI VFQ25, SF36 and depression screening questionnaires [29]

## Methods/design

This phase III single-masked randomised control study aims to determine whether there is a beneficial effect in using a slow-release dexamethasone preparation as an adjunctive treatment at the time of surgery in patients with established PVR following rhegmatogenous retinal detachment.

The 140 patients are to be divided into two equal groups (treatment and control arm). They are recruited for the study if they satisfy the inclusion and exclusion criteria. Participants are masked to their treatment allocation until completion of the study and the operating surgeon is masked until the time of IMP delivery.

Both groups will receive standard surgical treatment and routine preoperative and postoperative treatment and care comprising standard 3 port *pars plana* vitrectomy (or gel trimming if the eye was previously vitrectomised) and internal identification of retinal breaks; peeling of anterior and posterior epiretinal membranes and removal of subretinal membrane if required; relief of traction by retinectomy or placement of a scleral buckle at the operating surgeon’s discretion; retinopexy to retinal breaks and retinectomy edge by cryotherapy or laser; intraocular tamponade using 1300 or 5000 centistoke silicone oil. Intra-oil medication, the IMP, will be administered through a sclerostomy prior to wound closure in the treatment group.

A check for perfusion of the optic nerve head immediately after the injection of the IMP will be performed, along with digital indentation to confirm a satisfactory IOP once all scleral ports have been sutured. Biomicroscopy will be performed the following day to confirm the position of the implant.

If a patient is rendered aphakic as part of the operative procedure and they have been randomised to receive the IMP, the study treatment will not be given [30,31], but the patient will still be followed up as part of the study.

Removal of silicone oil (combined with cataract extraction plus intraocular lens implantation when applicable) will be planned for 3 to 5 months after primary surgery. On removal of oil, a second dexamethasone implant will be administered with performance of the same aforementioned safety monitoring. Again, if a patient is rendered aphakic or an anterior chamber intraocular lens is inserted as part of the operative procedure, and the patient has been randomised to receive the IMP, the study treatment will not be given. The patient will still be followed up as part of the study.

Patients who do not satisfy the criteria for controlled IOP preoperatively (see management of IOP), will not receive their second IMP injection.

### Study visits and assessment schedule

Postoperative study visits will not differ from the routine schedule for vitreoretinal procedures at the study site for the first 6 months that is day 1, day 10, day 30, 3 months and 6 months. Further assessments will be scheduled for 9 and 12 months post initial surgery. The time window allowed around these scheduled visits will be as follows: day 10 (+/- 3 days), day 30 (+/- 7 days) and months 3, 6, 9 and 12 (+/- 14 days). At each scheduled postoperative study visit, a full ophthalmic assessment will be completed, including slit lamp biomicroscopy (with indirect binocular ophthalmoscopy when required) and recording of parameters including ETDRS visual acuity, Goldman applanation tonometry, anterior segment assessment and retinal attachment status. SD-OCT will be used to record the central foveal thickness and total macula volume. An additional study visit to assess IOP has been included in the protocol schedule at day 60 postoperatively following both the study vitrectomy and the removal of oil procedure.

Silicone oil removal will not be considered a reoperation and there will be routine subsequent follow-ups until the patient can return to the study visit schedule. Other vitreoretinal interventions (with the exception of external retinal laser treatment, and macula ERM peel) over the trial period will be considered reoperations and recorded as such. Postoperative visits related to reoperations, or any other attendance outside the study visit schedule, will be recorded as 'unscheduled visits’. Case report forms (CRFs) identical in composition to the study scheduled visit CRF will be completed and included in the data analysis on completion of the study.

Following the final study visit at 12 months, participants will be discharged back to the care of their general practitioner. Participants requiring ongoing ophthalmic care will be followed up by their admitting consultant or will be under the care of a more appropriate specialist consultant, for example, a glaucoma specialist.

### Management of intraocular pressure

The commonest significant side effect of this dexamethasone preparation is raised intraocular pressure (≥ 25 mmHg) and was reported to occur in approximately 25% of participants in two phase III safety trials, peaking at day 60 and returning to baseline at 6 months. The investigators will include the additional risk of a rise in intraocular pressure with the trial treatment within the participant information leaflet (PIL).

As stated, an additional study visit at day 60 to assess IOP has been included in the protocol schedule and is specified in the PIL. The investigators will, as much as possible, adhere to an explicit management plan for raised IOP. This plan has been approved by an external glaucoma specialist and is given in Table 1.

**Table 1:** Algorithm for management of elevated intraocular pressure

**Intraocular pressure (mmHg)**	**Treatment**	**Follow-up**
≤ 25	None	As per protocol schedule
>25 but <30	Single topical ocular hypotensive	Within 6 weeks^*^
≥ 30 but <35	Dual topical ocular hypotensive	Within 6 weeks
≥ 35	Oral administration of acetazolamide 500 mg and dual therapy (recheck intraocular pressure within 2 hours)	After 2 hours:
		1. Intraocular pressure <35 mmHg – oral administration of Diamox 250 mg SR bd 5 days + dual topical therapy F/U 1 week
		2. Intraocular pressure ≥35 mmHg – same day glaucoma service input and/or consultant VR input

SR = slow release, bd = twice daily, F/U = follow up, VR = vitreoretinal.

^*^If intraocular pressure has not responded to single therapy or only partially responded, then a substitute agent will be tried or an additional agent added, respectively.

Patients will be referred to the glaucoma service if:

1. IOP remains >25 mmHg on dual therapy

2. Long-term IOP management is required (that is >2 consecutive months of IOP lowering agents required in the absence of an internal tamponade agent)

3. The investigators deem it in the patient’s interest

In the event where a patient’s IOP is >25 mmHg at the time of listing for the procedure to remove silicone oil, then additional topical ocular hypotensive agents may be started or added and the patient’s surgery postponed by up to 4 weeks until the IOP is ≤25 mmHg. Where the IOP remains >25 mmHg or if systemic ocular hypotensive agents are required to control the IOP, then patients randomised to receive the IMP will have their second injection omitted. This will be recorded and reported in the final manuscript.

### Eligibility

#### Inclusion criteria

1. All patients with established PVR (Grade C) following rhegmatogenous retinal detachment requiring surgery with planned silicone oil tamponade

2. Ability to give informed consent

3. Willingness to accept randomisation and attend follow-ups

Where the disease is bilateral at the time of screening, the worst affected eye (in terms of severity of PVR) will be included in the study.

#### Exclusion criteria

Individuals are excluded if they:

1. Are less than 18 years old

2. Have a history of open globe injury

3. Have a diagnosis of ocular hypertension and on two or more pressure-lowering medications

4. Have a definite diagnosis of glaucoma and if in the opinion of a glaucoma specialist, the patient is at high risk of visual damage from raised IOP

5. Have uncontrolled uveitis

6. Have previous steroid-induced glaucoma

7. Have proliferative diabetic retinopathy

8. Are pregnant or breastfeeding females (females with child-bearing potential must have had a negative pregnancy test within 7 days of commencing the trial and agree to use adequate contraception throughout the duration of the trial)

9.  Have had a previous known adverse reaction to the IMP

10.  Have a suspected ocular or periocular infection (for example, Herpes Simplex Virus, Varicella Zoster Virus, mycobacterial or fungal disease)

11.  Are aphakic or if a lensectomy is planned at time of surgery

12.  Have a pre-existing anterior chamber intraocular lens

### Recruitment and randomisation

Patient recruitment will only be done when the trial has documented research and ethics committee (REC), regulatory and local trust R&D approval. The study is conducted in accordance with the International Conference on Harmonisation for Good Clinical Practice, as set out in the European Union Clinical Trials Directive (2001) and associated UK Regulations (2004). The study will comply at all times with the Declaration of Helsinki (2000).

All 140 participants will be identified and recruited from outpatients and emergency referrals at Moorfields Eye Hospital. At screening, a structured interview will be conducted by research staff, which will include questions on coexisting ocular pathology and previous ophthalmic surgical procedures to confirm that all inclusion and exclusion criteria are satisfied.

Patients are then randomised to either the treatment arm or control arm. The randomisation list was generated using permuted blocks of varying sizes and was generated by a senior data manager independent of the trial team. The list of 140 study IDs will be held by the trial pharmacist, and following informed consent and recruitment into the trial, participants will be allocated to the lowest unused study ID. Out of hours (that is, weekends and bank holidays) when access to the trial pharmacist is limited, the next study ID in sequence will be kept in a sealed envelope in a secure location on site.

### Screening and baseline assessment

An initial screening assessment will be performed prior to recruitment to confirm that patients satisfy all inclusion and exclusion criteria. This will include a full ophthalmic examination with slit-lamp/indirect biomicroscopy and consider the patient’s medical history and concomitant medication.

Clinical findings documented as part of the routine clinical care at the time of screening may be used to populate data in the baseline CRF and used as part of the study data (if collected within 1 week of the study vitrectomy). This information may be collected prior to informed consent for enrolment into the trial as no additional intervention is performed outside routine clinical care.

Baseline assessments will be performed within 1 week of the scheduled operation date. This will include: patient demographics, past ocular history, logMAR visual acuity (ETDRS method), slit lamp/indirect ophthalmic examination (anterior and posterior segment assessment, lens status, extent of retinal detachment, grade of PVR [32]), SD-OCT-guided foveal thickness and volume, and quality of life questionnaires.

### Masking

Participants are masked to their treatment allocation until their completion of the study. The operating surgeon is masked until the end of the procedure just prior to sclerostomy closure, to avoid any bias regarding surgical management. It is not possible to mask the investigators actively as the IMP may be visible during posterior chamber assessment until its degradation. A placebo vehicle was not used as a comparator as it was deemed unethical due to lack of safety data, and the scientific justification is that the treatment group can be compared to the standard care group.

### Outcome measures

#### Primary

The primary outcome will be whether there is stable anatomic reapposition of the retina to the retinal pigment epithelium without additional vitreoretinal intervention in the absence of an internal tamponade agent at 6 months post study vitrectomy.

#### Secondary

Secondary outcomes will be performed at 6 and 12 months following the primary study vitrectomy:

•visual acuity: a comparison of the median visual acuity and the proportion of patients in each group achieving a visual acuity of 55 ETDRS letters or better

•macula oedema and thickness (SD-OCT analysis), that is the proportion of patients in each group with a central A1 macula subfield measure of >300 μm

•the proportion of patients in each group who develop overt PVR recurrence

•the proportion of patients in each group achieving complete retinal reattachment

•the proportion of patients in each group achieving stable posterior (post equatorial) retinal reattachment

•the proportion of patients in each group with a tractional retinal detachment

•the proportion of patients in each group who suffer hypotony (defined as IOP <6 mmHg and/or raised IOP (defined as >25 mmHg) at any time during the study period

•the proportion of patients in each group who develop macula pucker or epiretinal membrane (ERM) and/or require macula ERM surgery at any time during the study

•the proportion of patients in each group who require cataract surgery at any time during the study

•quality of life assessment: a comparison of the median or mean scores of both SF36 and VFQ25 between both groups and the proportion of patients with severe depression [29]

### Adverse events and safety reporting

Safety reporting will adhere to the sponsor’s standard operating procedures and the trial team are confident that means are in place to monitor, record and report adverse events in line with guidelines issued by the Medicines and Healthcare Products Regulatory Agency (MHRA). There is an external data monitoring committee, who have an agreed charter. They will meet six to twelve monthly or on an *ad hoc* basis as required.

Expected adverse events are as follows:

1. Cataract

2. Raised IOP

3. Hypotony

4. Sterile hypopyon

5. Retinal detachment

6. Uveitis

7. Further surgery

8. Glaucoma

9. Headache

10. Migraine

11. Vitreous opacities

More specifically, the recording of the severity of raised IOP will be as follows:

Mild: >25 mmHg and <35 mmHg

Moderate: ≥35 mmHg

Severe: Any interventional invasive procedure (for example, surgery or laser treatment) required to control the elevated IOP acutely or long-term, during the study period

Unexpected adverse events will include:

1. Endophthalmitis

2. Systemic illness

3. Ocular vascular occlusion

4. Other

### Trial size and timescale

Based on the results of the primary outcome measure from a trial of the same patient group carried out in the study centres [7], 66 patients per study arm are required for a study power of 85% to detect, at the 5% level, a 50% improvement in the success of the adjunctive regime (reducing failure from 49% to 24%). A 50% reduction in failure would represent a marked clinical benefit and would be likely to be adopted on a large scale by vitreoretinal surgeons. Our pilot study has shown that this is a realistic treatment difference. Given a 5% loss to follow-up and protocol violations (previous similar studies at Moorfields had rates of 3% to 5% loss to follow-up or protocol violation [7-9]), this gives a study total of 140 patients to be randomised.

### Proposed timescale

Trial start: February 2012

Projected trial end: February 2015

Trial duration: 36 months

Duration of each patient’s participation: 12 months

### Statistical analyses

The statistical analysis plan will be written in advance of the data analysis by the trial statistician and will be approved by the trial steering committee. Analysis will be by intention to treat (ITT) to retain validity of the randomisation process. The trial ITT population comprises all randomised patients regardless of eligibility (inclusion/exclusion) errors, post randomisation withdrawal and whether the correct study treatments were received. Data analysis will adhere to the CONSORT guidelines for randomised controlled trials. The baseline characteristics of the two groups will be compared to assess the adequacy of randomisation.

### Primary endpoint analysis

The chi-square test will be used to assess the statistical significance of any observed differences between the proportions of patients who 'fail’ as defined by the primary outcome measures at 6 months in the two groups. Logistic regression will be conducted to assess for any baseline covariates believed to be associated with the outcome.

### Secondary endpoint analysis

Summary statistics will be computed for changes in visual acuity for the two treatment groups at 6 and 12 months and the proportion of patients in each group who suffer any adverse events will be summarised. The exact binomial method will be used to compute 95% confidence intervals. Summary statistics for all other secondary outcomes will be provided by treatment group and if statistical comparisons are made, results will be reported as exploratory.

### Missing data

The sample size calculation assumed 5% of patients would not provide an evaluable 6 month outcome. If data are missing for any patients, reasons for missing data may be important and these will be examined using logistic regression of covariates on an indicator of missing data. An available case analysis will be conducted together with an ITT analysis (using imputation) and results compared in a sensitivity analysis. Any deviations from the statistical analysis plan will be described and justified in the final report, as appropriate.

### Trial organisation and monitoring

#### Trial management committee

David Charteris: Chief investigator, Moorfields Eye Hospital, London, UK

Philip Banerjee: Co-investigator, Moorfields Eye Hospital, London, UK

Catey Bunce: Co-investigator and statistician, Moorfields Eye Hospital, London, UK

Rachel Yoon: Trial pharmacist, Moorfields Eye Hospital, London, UK

Nicola Harris: Trial manager, Moorfields Eye Hospital, London, UK

T. Margaret Zvobgo: Research nurse, Moorfields Eye Hospital, London, UK

#### Trial steering committee

Professor Roger Hitchings: Chair, Emeritus Professor of Glaucoma and Allied Studies, University College London and Moorfields Eye Hospital, London, UK

Hadi Zambarakji: Consultant ophthalmologist and clinical trialist, Whipps Cross University Hospital NHS Trust, London, UK

Sue Beer: Lay person

David Charteris: Chief investigator, Moorfields Eye Hospital, London, UK

Philip Banerjee: Co-investigator, Moorfields Eye Hospital, London, UK

Catey Bunce: Co-investigator and trial statistician, Moorfields Eye Hospital, London, UK

#### External data monitoring committee

Timothy Jackson: Consultant vitreoretinal surgeon and clinical trialist, King’s College London, UK

Professor Robert Maclaren: Nuffield Laboratory of Ophthalmology and Oxford Eye Hospital Biomedical Research Centre, University of Oxford, John Radcliffe Hospital, Oxford, UK

Victoria Cornelius: Senior lecturer in medical statistics, King’s College, London, UK

David Broadway: Consultant ophthalmologist, and glaucoma specialist, Norfolk and Norwich University Hospitals NHS Foundation Trust, UK

### Trial documentation and data collection

The CRFs will be designed and produced by the investigators, according to the sponsor’s CRF template. The final version will be approved by the sponsor. It will be the responsibility of the investigators to ensure the accuracy of all data entered onto the CRFs. A delegation log will identify all trial personnel with responsibilities for data collection and handling, including those who have access to the trial database. Data handling will adhere to the Data Protection Act, 1998.

### Ethics and competent authority review

Applications to the UK’s main REC (NRES Committee London – Central) and the local Moorfields Research Management Committee have received favourable opinions and a Clinical Trial Authorisation has been issued by the MHRA.

### Publication policy

The results of this study will be submitted for publication in peer-reviewed medical journals regardless of whether the findings are in favour of the trial intervention.

## Discussion

This randomised controlled trial investigating the use of a sustained-release dexamethasone implant will be the first to evaluate its role as an adjunctive agent, in patients undergoing vitreoretinal surgery for retinal detachment with established PVR.

Our projected recruitment rate is based on a retrospective audit of the incidence of relevant cases at the study site. Due to the poor prognosis associated with current standard treatment, we expect a high recruitment uptake following successful eligibility screening. We remain optimistic that our recruitment target will be met within the projected timescale.

We accept that although our inclusion criteria specifies Grade C PVR requiring surgery with silicone oil, the cohort of participants is likely to be quite a heterogeneous group. The severity of Grade C PVR can vary widely throughout its sub-classification both in terms of extent (number of clock hours) and distribution (focal, diffuse, anterior, posterior or subretinal). However, we expect the adequacy of randomisation to compensate for this and shall acknowledge any unequal weighting within the groups as limitations of the study. We do, however, accept that we may limit our sensitivity to small differences between the two groups.

As this single centre study serves a wide geographical catchment area with a broad patient demographic representative of the UK, we do not expect the results to provide a misleading estimate of treatment effect. Again, we expect the adequacy of randomisation to compensate for any variation of effect between ethnicities.

The authors acknowledge the limitations of a single masked study, but have made every effort to reduce investigator bias. As the IMP is no longer visible inside the eye after approximately 8 weeks, it is unlikely that the investigators will be aware of the treatment allocation of the participant at the time of the primary and secondary outcome assessments. The trial team considered using an independent assessor of the primary outcome measure at 6 and 12 months, but felt that there was insufficient evidence to suggest this was necessary and that it added additional unjustifiable cost to the study.

Furthermore, by 1) masking the operating surgeon to the treatment allocation until the end of the surgical procedure and 2) explicitly defining the clinical findings and adverse events and adhering to rigid management protocols, (for example, for an IOP rise), we have limited investigator bias as much as possible.

In summary, this is the first randomised controlled clinical trial to investigate the use of an adjunctive slow-release dexamethasone implant in patients undergoing vitrectomy surgery for retinal detachments with proliferative vitreoretinopathy.

## Trial status

The authors confirm that the trial was in active recruitment at the time of manuscript submission.

## Abbreviations

CRF: Case report form; IMP: Investigational medicinal product; IOP: Intraocular pressure; ITT: Intention to treat; MHRA: Medicines and healthcare products regulatory agency; PIL: Participant information leaflet; PVR: proliferative vitreoretinopathy; REC: Research and ethics committee; SD-OCT: Spectralis domain optical coherence tomography; ETDRS: Early treatment diabetic retinopathy study; ERM: Epiretinal membrane.

## Competing interests

The authors declare that they have no competing interests.

## Authors’ contributions

PJB participated in development of the trial protocol, gained regulatory authority approvals, coordinated the trial set-up at the study site, prepared the standard operating procedures, study documentation and drafted the manuscript. CB participated in development of the trial protocol, standard operating procedures, study documentation and contributed to drafting the manuscript. DGC conceived and designed the trial, secured trial funding, gained regulatory authority approvals, prepared the trial set-up, prepared the standard operating procedures, study documentation and contributed to drafting the manuscript. All authors read and approved the final manuscript.
